# Costs are a major driver of antibacterial drug prescriptions in Germany: market analysis from 1985 to 2022

**DOI:** 10.1007/s00210-024-03171-y

**Published:** 2024-06-06

**Authors:** Lilly Josephine Bindel, Roland Seifert

**Affiliations:** Institute of Pharmacology, Hannover Medical School, D-30625 Hannover, Germany

**Keywords:** Antibacterial drug consumption, Antibacterial drug prescription, Germany, Antibacterial resistance, Arzneiverordnungsreport, Antibacterial stewardship

## Abstract

In recent years, several threatening developments regarding antibacterial drugs, such as rising bacterial resistance and delivery bottlenecks, have occurred. Since antibacterial drugs are crucial for modern medicine, understanding events and influencing factors relevant for long-term developments is essential. Therefore, we analyzed the number of prescriptions and costs, defined daily dose (DDD) and DDD costs of antibacterial drugs in Germany, based on the Arzneiverordnungsreport (AVR, Drug description report) between 1985 and 2022. Based on prescription rates in 2022, we selected the TOP15 and TOP5. For a more in-depth analysis, we analyzed data from the wissenschaftliches Institut der AOK (WidO, scientific institute of the AOK). The number of prescriptions increased between 1985 and 2013, but since 2014, there has been a declining trend with a noticeable COVID-related dip. Over the years, a shift in drug classes occurred. Once very popular drugs like penicillins and tetracyclines are no longer as important. Conversely, aminopenicillins and cephalosporins have become more relevant. Particularly, the TOP5 drugs have seen an increasing proportion. DDD costs have decreased in most substance classes over time. There is a strong association between decreasing costs and rising prescriptions. Falling costs have a stronger immediate impact on prescriptions that rising costs. When costs are very low, drugs might be prescribed excessively. Supply bottlenecks can also result. The main prescribers are mainly in primary care. Their share of prescription has changed little over the years, but is decreasing regarding total consumption. In comparison to other European countries, Germany ranks in the lower third regarding prescriptions. In most countries, the COVID pandemic has led to a reduced prescription of antibacterial drugs. In conclusion, we provided a comprehensive overview of the antibacterial drug market in Germany over the past four decades and identified costs as a major driver of antibacterial drug prescriptions. Increased costs may reduce uncritical prescription of antibacterial drugs, development of bacterial resistance, supply shortages and occurrence of adverse effects.

**Supplementary Information:**

The online version contains supplementary material available at 10.1007/s00210-024-03171-y.

## Introduction

Every year, the prescriptions and costs for drugs regarding the GKV in Germany are published in the Arzneiverordnungsreport (AVR, Drug prescription Report). Current data, trends and important changes are presented. Although developments in recent years are discussed, the analysis never goes back further than 10 years. The current situation in Germany is characterized by declining consumption of antibacterial drugs in recent years (ECDC 2023a). Nevertheless, there are increasingly serious problems such as rising bacterial resistance (DART2030 2023) and frequent supply bottlenecks (BfArM 2023). Similar problems can also be observed in other countries of the European Union.

It is essential to get an overview of the entire development, especially for important drugs like antibacterial drugs (Laxminarayan et al. 2013). Therefore, we examined the development of prescriptions and costs since the beginning of recording in 1985. The aim is to analyze developments and to identify influencing factors. It is important to understand the effects of measures taken in the past to make appropriate decisions in the present, enabling us to react to challenges such as bacterial resistance or supply bottlenecks.

## Materials and methods

### Data collection

**Fig. 1:**
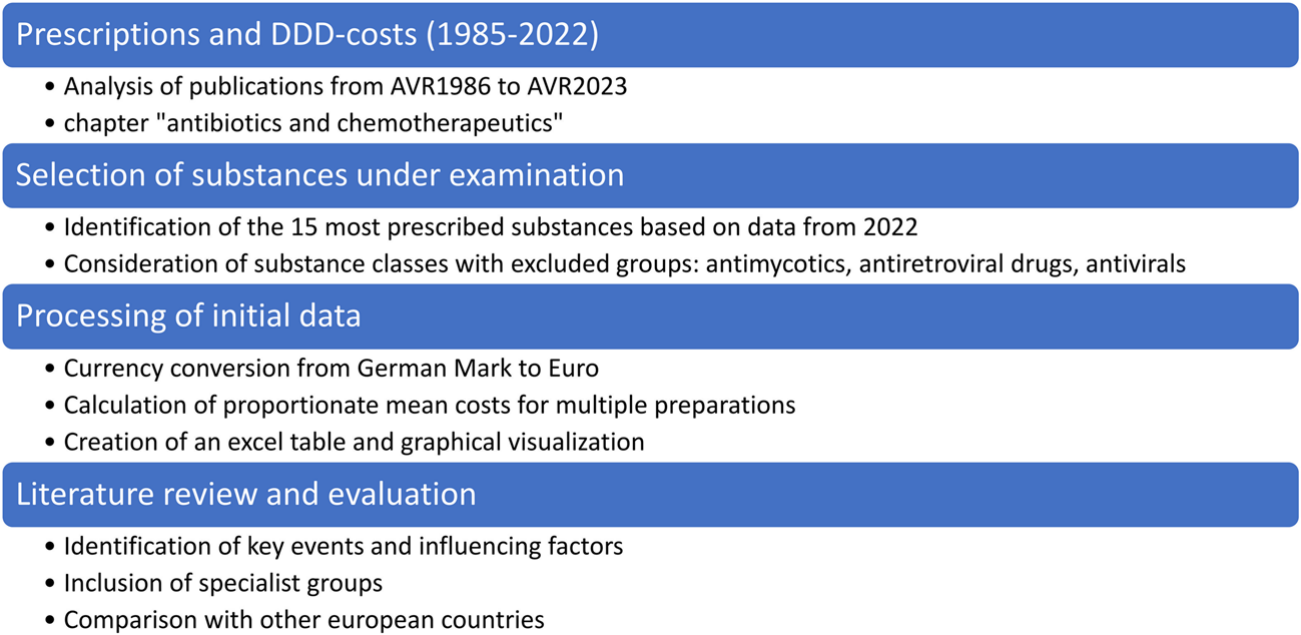
Procedure for analysing prescription data of antibacterial drugs

In the following analysis, based on the Arzneiverordnungsreport (AVR, Drug prescription report) from 1985 to 2023, we considered prescriptions and defined daily dose (DDD)-costs of antibacterial drugs (Schwabe and Paffrath, 1985, 1986, 1987, 1988, 1991, 1992, 1993, 1994, 1995, 1996, 1998, 1999, 2000, 2001, 2002, 2003, 2004, 2006, 2007, 2008, 2010a, 2010b, 2011a, 2011b, 2012, 2013, 2014, 2015, 2016, 2017; Schwabe, Paffrath and Anlauf 1989, 1990; Schwabe, 1997; Schwabe, Paffrath, Ludwig and Klauber, 2018, 2019; Schwabe and Ludwig, 2020; Ludwig, Mühlbauer and Seifert, 2021, 2023, 2024). Since we focused on outpatient prescriptions, only the general chapter “Antibiotika und Chemotherapeutika” (antibiotics and chemotherapeutics) was considered. Therefore, specialized subchapters like urology, dermatology and ophthalmology were excluded. Figure 1 shows the analytical procedure of our study.

### Selection of drugs

For a general overview, we collected data from categorized substance classes. We only included antibacterial drugs, resulting in the exclusion of antimycotics and antivirals. Particularly interesting were the most prescribed drugs, for which we selected the TOP15 based on the year 2022.

### Analysis of specialist groups

For a deeper understanding of which medical fields have the main share of consumption, we analyzed the data provided from the WidO. These data are consistent with the data from the Arzneiverordnungsreport. Records are available from 2008 to 2022. The years 2008 to 2018 were kindly provided to us directly by WidO, while the years 2019 to 2022 are publicly available (WIdO 2020, 2021, 2022, 2023). We focused on the main prescribers and on the development of the relative shares.

### Preparation, evaluation and presentation of data

After collecting data from all years, in some cases, processing initial data was necessary. This included currency conversion from the German Mark to the Euro and calculating proportionate mean costs when several preparations were depicted.

Once all the data were available in a uniform format, the Excel tables were visualized. Literature review was conducted to identify key events and influencing factors, allowing us to link the curves to real events. Figures 2, 3, 4, 5, 6, 7, 8, 9, 10, 11, 12, 13, 14, 15 and Table 1 show the most important results of our study. Supplemental Figures S1-S8 present some more detailed analyses of the dataset.

**Fig. 2:**
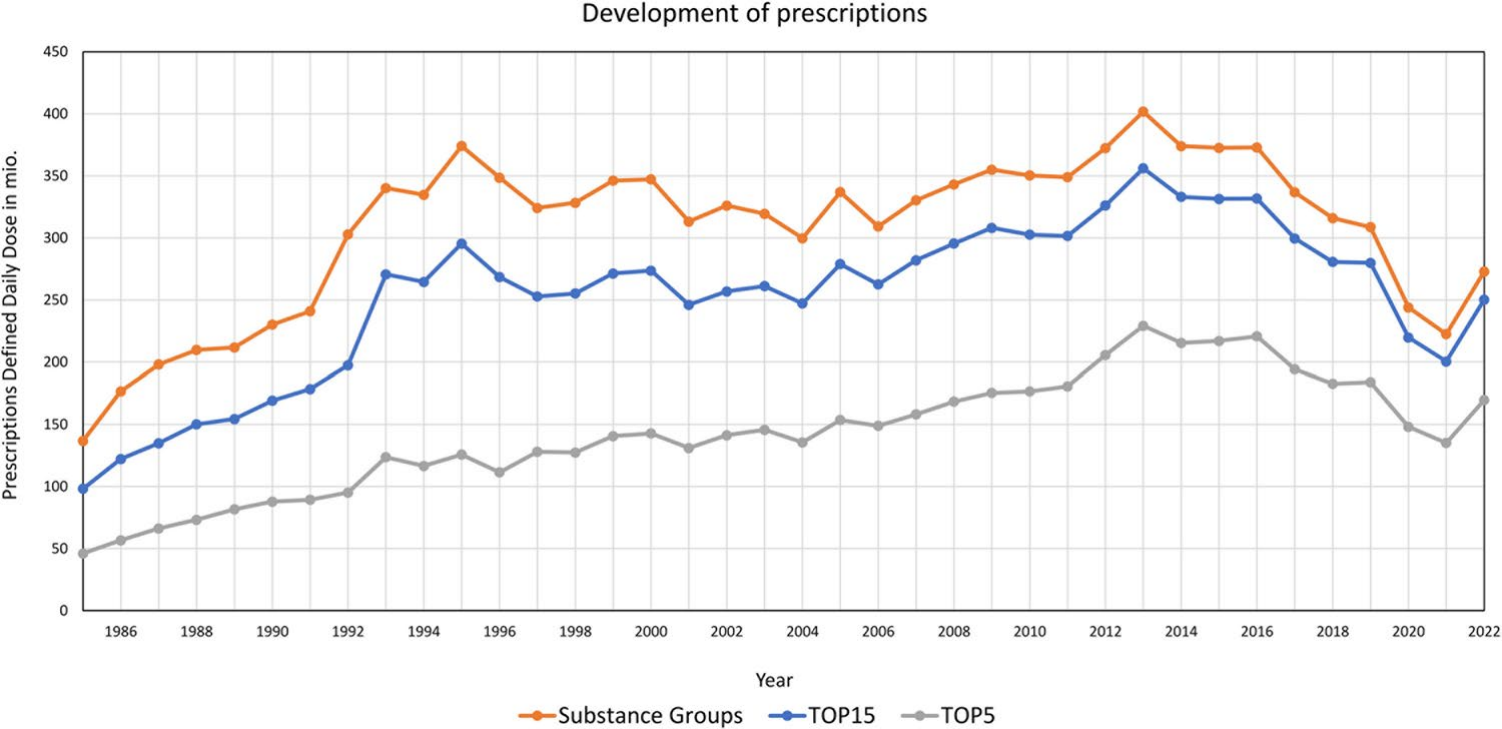
Development of prescriptions Defined Daily Dose in millions (1985–2022)

## Results

### Prescriptions defined daily dose

Comparing 1985 and 2022, the number of prescriptions increased in total as well as for the most frequently prescribed 15 drugs (TOP15) and 5 drugs (TOP5) (Fig. 2). Between 1985 and 1995, the prescriptions rose particularly sharply. It is not possible to specify the increase in consumption to a specific event. Several political and sociological factors could have played a role. In addition to the Yugoslav Wars, the German reunification also occurred. Prior to 1990, data was only available for the Western part of Germany. In 1990, the consumption of the former Eastern part of Germany was 56.5 million DDD (Schwabe and Paffrath 1991) . Following this, data from both the Western and Eastern part of Germany were included in the analysis. After that, a plateau formed in the following years. Following a peak in 2013, there was a decline in prescriptions from 2014 to the present. A distinct COVID-related anomaly is evident in 2020 and 2021 (Selke Krulichová et al. 2022).

**Fig. 3:**
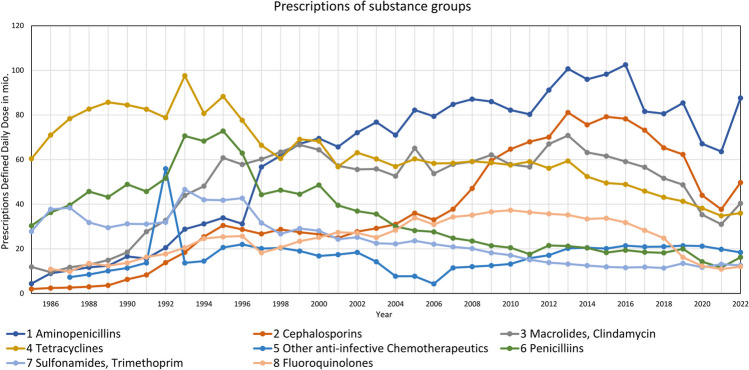
Development of prescriptions Defined Daily Dose in analysed substance groups

In all substance classes, changes occurred (Fig. 3). Aminopenicillins showed a continuous increase with a strong shift upwards in 1997. In contrast, penicillins are decreasing since 1997. Cephalosporins showed a first increase followed by a plateau from 1990 and a second even sharper rise from 2007, but this was followed by an ongoing drop. Macrolides became relevant since 1990 and reached a plateau since 1995. Tetracyclines were the most prescribed group in 1985, but since the late 1990s, their importance decreased. Other antibacterial drugs show a slight, unsteady increase. Sulfonamides were very popular in the 1990s but were then prescribed less with a further decreasing trend. Fluoroquinolones showed a slow increase till 2010, after that, an ongoing shrinkage began. Since around 2017, nearly all groups show a decrease. Apart from this, there is a sharp drop in the number of prescriptions in 2020 and 2021.

**Fig. 4:**
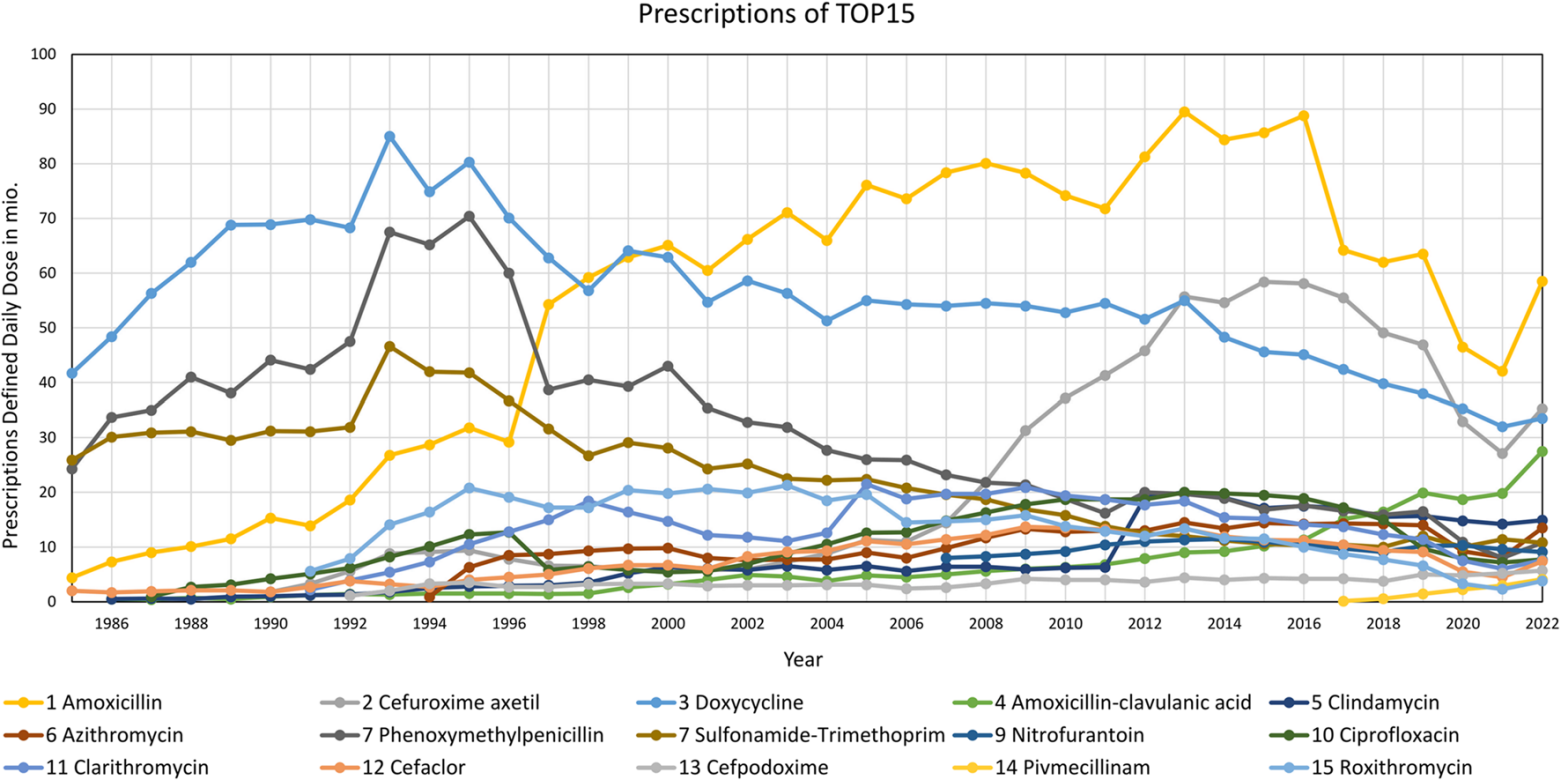
Development of prescriptions Defined Daily Dose in TOP15 antibacterial drugs

The TOP5 show very different courses (Fig. 4). Amoxicillin showed a strong step upwards in 1997, followed by a further increase with a peak in 2012. A sharp drop is recognisable in 2016. Ignoring the dip in 2020 and 2021, it remains on a plateau since then. Cefuroxime axetil showed a very fast and strong increase since 2007, followed by an equally rapid fall from 2016. Doxycycline was very dominant at the beginning with a peak in 1993. Since the late 1990s, a decrease started. Amoxicillin-clavulanic acid is rising since the start of recording. Clindamycin slightly increased from 1989 till 2000, followed by a plateau and a strong upward leap in 2011. After that, a steady decrease is recognizable.

In contrast to the TOP5, all the TOP6-10 show a decreasing trend. Phenoxymethylpenicillin and sulfonamide-trimethoprim were very popular in the 1990s, followed by an ongoing decrease. Azithromycin, nitrofurantoin, and ciprofloxacin have a relatively constant trend in their prescription volume.

Nearly all TOP11-15 have a decreasing trend or a plateau on a low level. One exception, however, is pivmecillinam. It was launched in 2016, since then, it shows a quick linear increase in prescriptions.

**Fig. 5:**
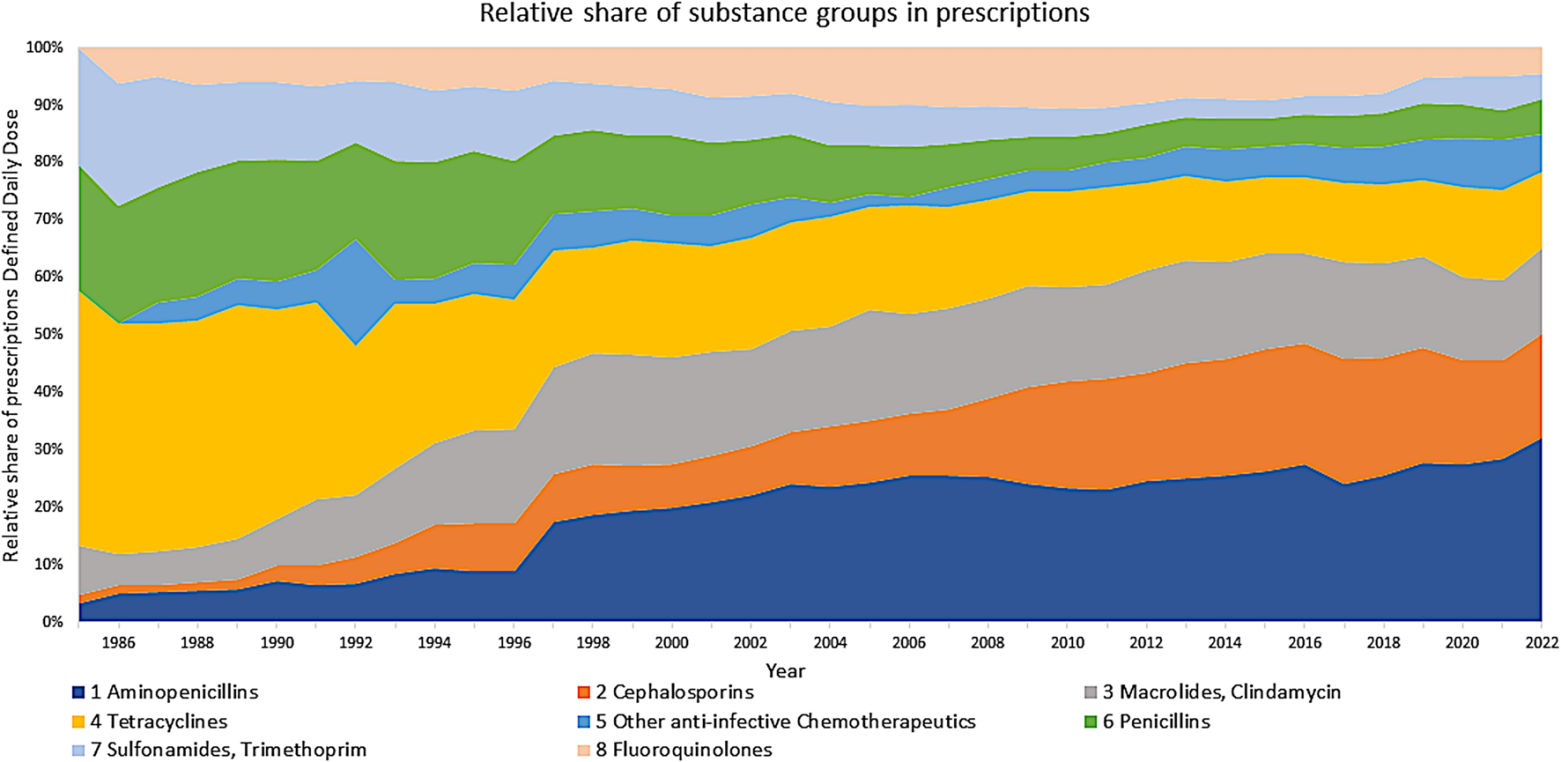
Development of dominance in substance groups, shown as relative share in prescriptions Defined Daily Dose

In addition to the total development of prescriptions, the relevance of the groups can be measured by their relative share (Fig. 5). Aminopenicillins show an increasing importance. The second most frequently prescribed group, cephalosporins, showed an increasing share until 2017, but decreased since then. The third group, macrolides, has a comparable progression as cephalosporins. Tetracyclines, penicillins and sulfonamides have lost shares over the years. Fluoroquinolones became quite popular in the early 2000s but are losing importance since then.

**Fig. 6:**
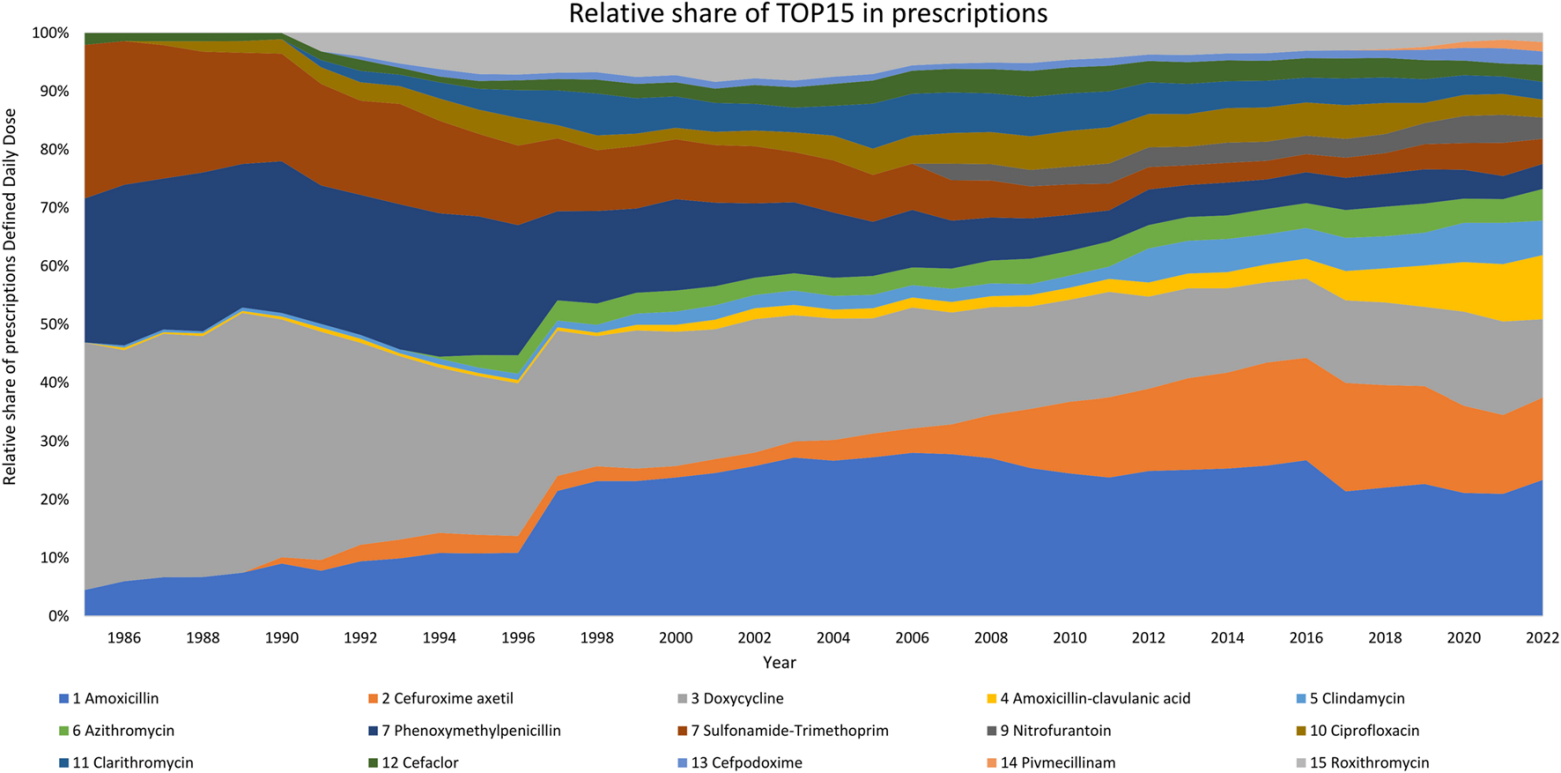
Development of dominance regarding individual antibacterial drugs (TOP15) on a relative share inprescriptions Defined Daily Dose

As with the substance classes, strong changes in prescription within the TOP15 antibacterial drugs are seen (Fig. 6). Once very popular drugs like phenoxymethylpenicillin and sulfonamide-trimethoprim lost a lot of shares. Other drugs like roxithromycin and clarithromycin were popular for a while but are losing significant shares since then. Slowly increasing proportions can be recognised for amoxicillin-clavulanic acid, clindamycin, azithromycin, nitrofurantoin cefpodoxime and pivmecillinam. Amoxicillin has a relatively constant share after an increase in 1997.

### DDD-costs

**Fig. 7:**
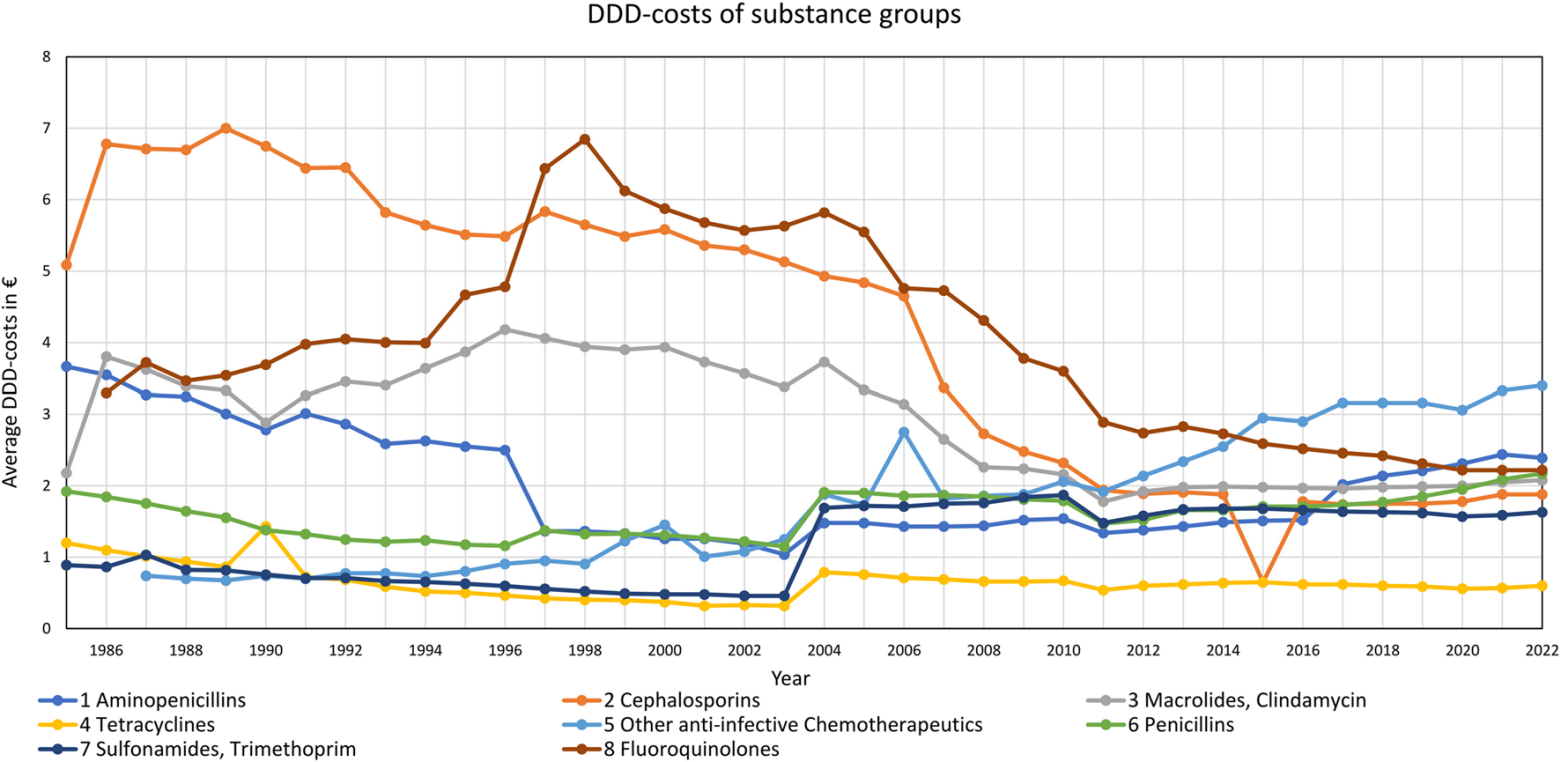
Development of average DDD-costs in substance groups (1985–2022)

In most of the substance groups, DDD-costs fell (Fig. 7). Large price reductions took place until 2011. After that, DDD costs approached a plateau with slight increases in recent years. This applies to aminopenicillins, cephalosporins, macrolides, tetracyclines and fluoroquinolones. On the contrary, the average DDD-costs of other anti-bacterial drugs, i.e., penicillins and sulfonamides rose.

**Fig. 8:**
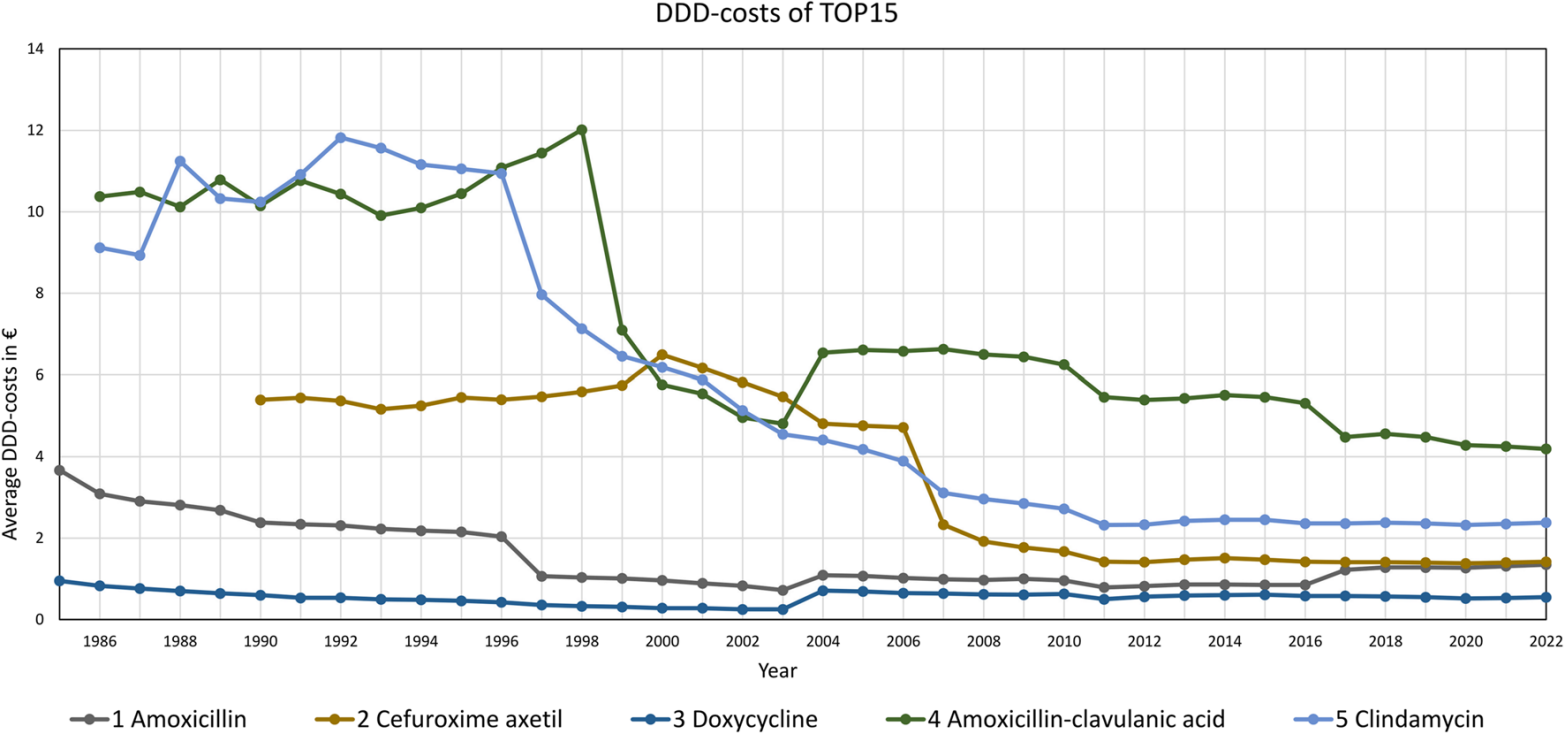
Development of average DDD-costs in the TOP15 prescribed antibacterial drugs (1985–2022)

At the level of individual drugs, average DDD-costs are falling for almost all preparations. In Fig. 8, this is depicted for each drug. Few exceptions are sulfonamide-trimethoprim and pivmecillinam with slightly higher costs than at the beginning. In 2004, an increase can be observed across all preparations. Most price reductions took place up to 2011. After that, the costs of the TOP15 drugs remained more or less constant.

### Specialist groups

**Fig. 9:**
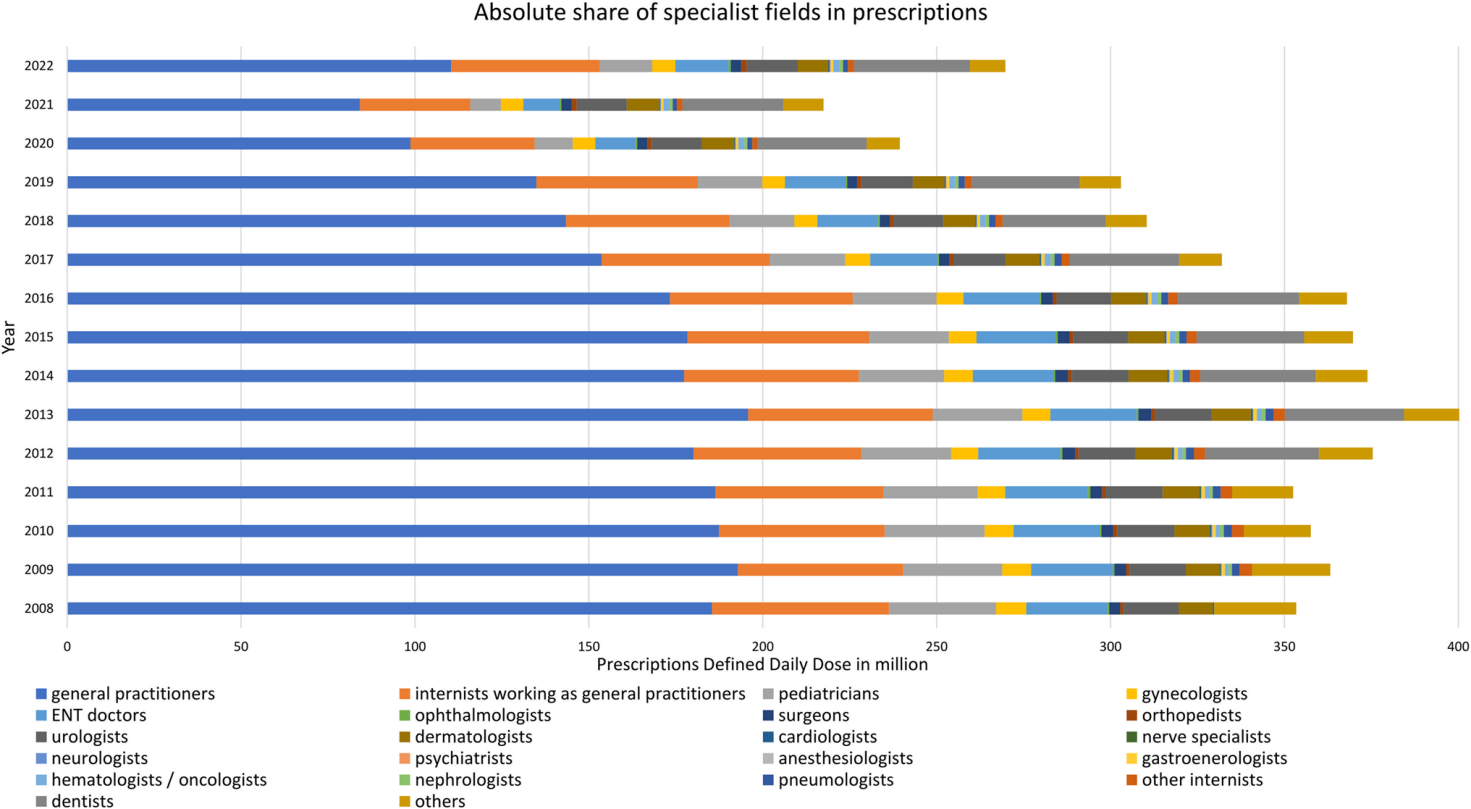
Total prescriptions of antibacterial drugs from 2008–2022, broken down by specialist groups

Regarding the different medical fields, prescriptions developed in line with the total prescriptions. Between the years, there were only slight changes in the individual groups. Total prescriptions decreased from 2008 to 2022 (Fig. 9). There was a particularly sharp drop in nearly all specialist groups in 2020 and 2021. Prescriptions rose again slightly in 2022.

Primary care settings, such as general practitioners or internists, have the highest prescriptions. Specialized fields, like cardiology or neurology, accounted for a smaller proportion of prescriptions. Prescriptions decreased for pediatricians, gynecologists, ENT doctors, surgeons, urologists, dermatologists, cardiologists, nerve specialists, anesthesiologists, gastroenterologists, pneumologists and other internists. Conversely, rising prescriptions were observed for ophthalmologists, orthopedists, neurologists, psychiatrists, hematologists/oncologists and dentists. Nephrologists maintained consistent prescription level throughout the analyzed period.

**Fig. 10:**
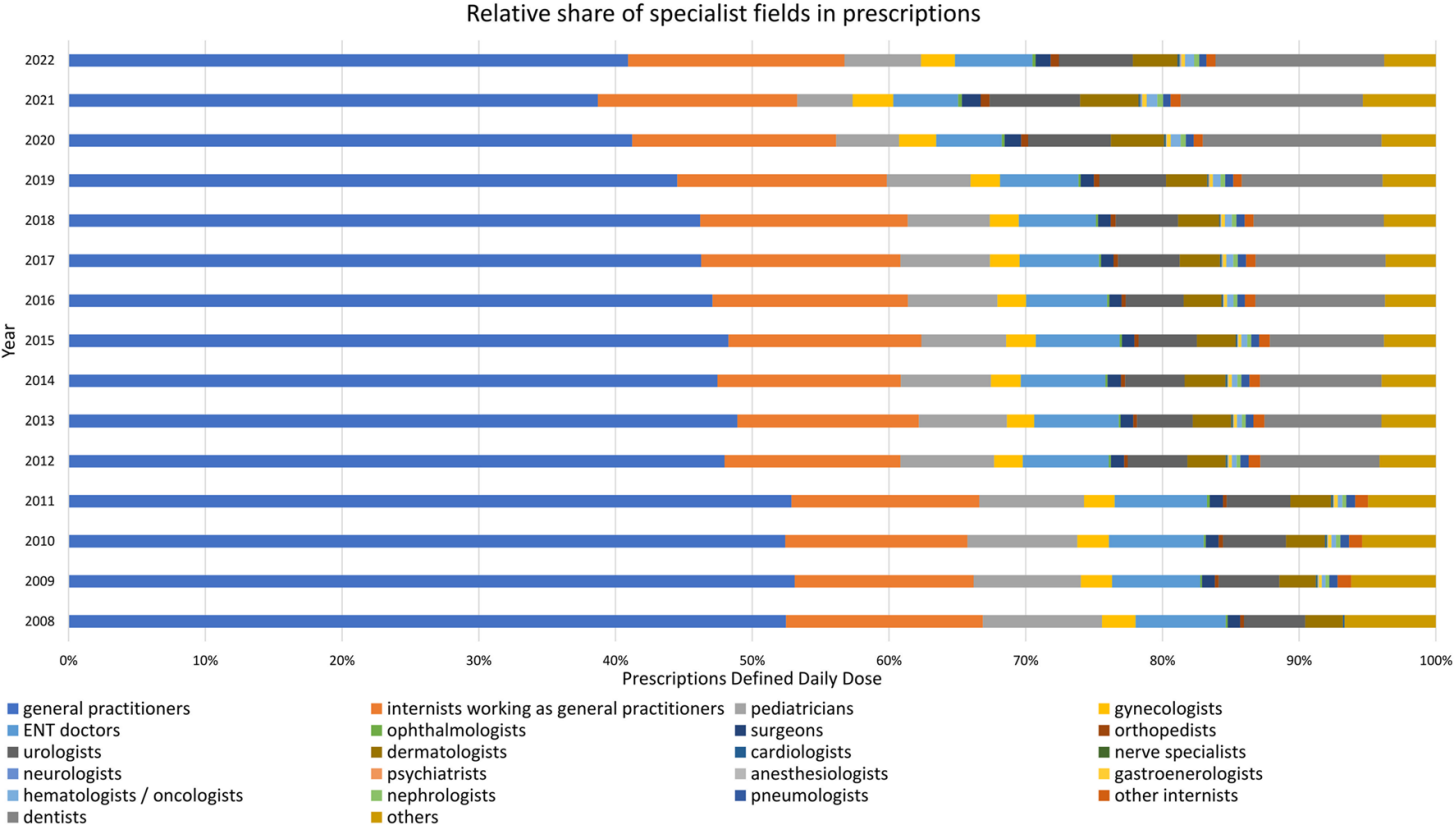
Relative prescriptions of antibacterial drugs from 2008–2022, broken down by specialist groups

The distribution of shares among specialist groups has remained relatively consistent throughout the period under review. Upon initial examination on Fig. 10, there appears to be a shift from 2012, but this can by explained by the inclusion of dental prescriptions. In Fig. S8, dentists were excluded. There, the proportion of dominant groups remains largely the same. In outpatient settings, a few groups are prescribing the main share. In 2022, general practitioners, internists working as general practitioners and dentists emerged as the primary prescribers. Conversely, nerve specialists, psychiatrists and anesthesiologists prescribed minimal quantities of antibacterial drugs.

## Discussion

There has been a shift in dominant drug classes over the past four decades. Once very popular drugs like penicillins and tetracyclines are no longer as important. Conversely, aminopenicillins and cephalosporins have become more relevant. At the level of individual drugs, very dominant and less significant drugs are distinguishable. Particularly, the TOP5 drugs have seen an increasing proportion. Why prescriptions in general were sinking since 2014, is not fully comprehensible. A reason could be an awareness of the importance of sparing use.

In some years, cross-drug events are noticeable. In 1997, the sharp rise in amoxicillin triggered a significant decline in almost all other preparations and groups (see Figs. 3 and 4). In 2004, the sharp rise in DDD-costs of many drugs caused a short-term downturn in prescriptions (see Fig. 2). In 2020 and 2021, the COVID pandemic caused a sharp drop. This was induced by far-reaching contact restrictions, resulting in a reduction in the opportunities for pathogenic bacteria to spread and consequently less infections. This especially applies to respiratory tract infections (Selke Krulichová et al. 2022). In 2022, following the COVID pandemic, the number of prescriptions approached pre-pandemic levels.

### Development of substance groups and TOP15

#### Aminopenicillins

**Fig. 11:**
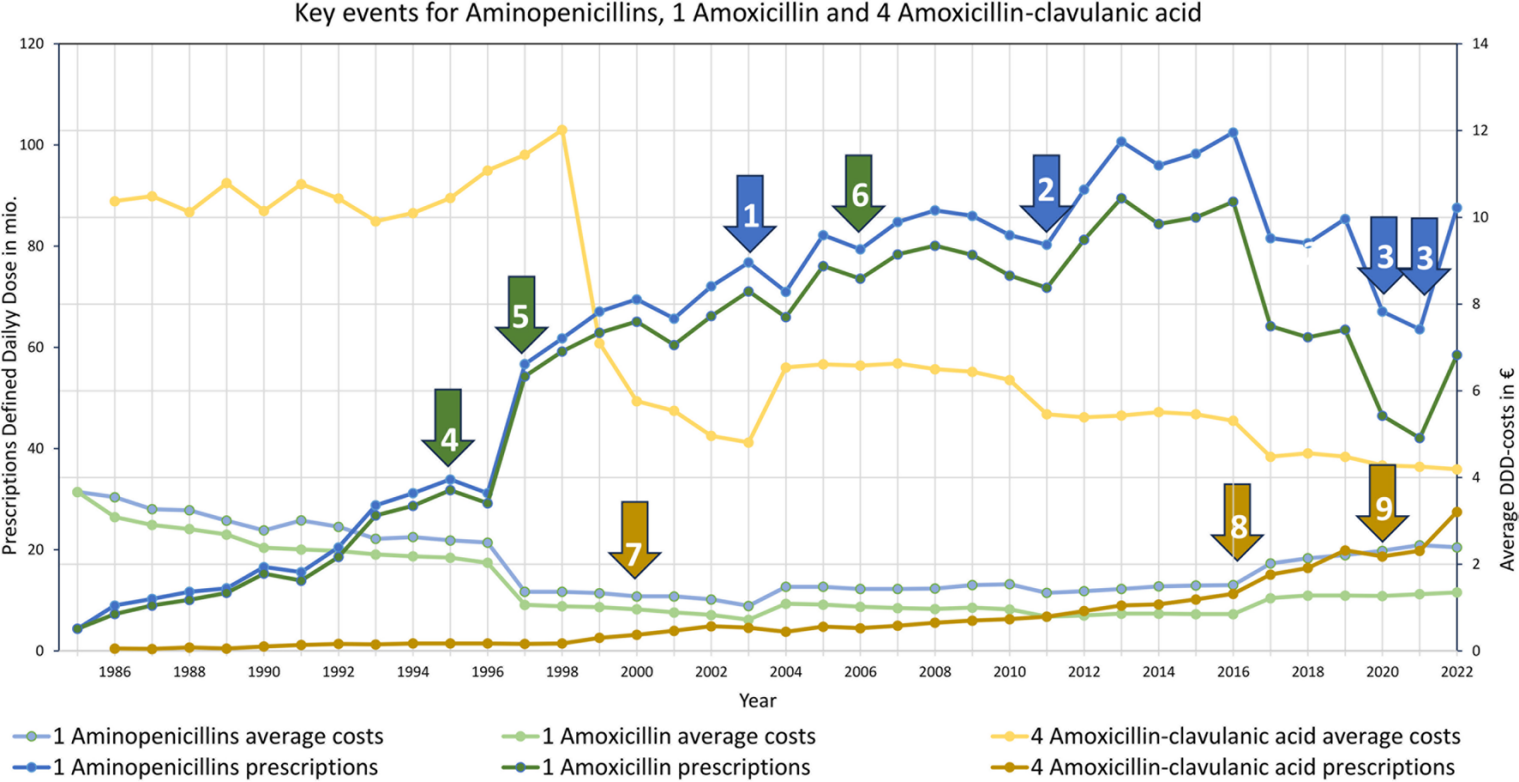
Key events for aminopenicillins in general are (1) lower costs than penicillins, (2) distortion due to the inclusion of dental prescriptions, (3) COVID-pandemic. Key events for amoxicillin are (4) therapy component in the treatment of gastric ulcers caused by H. pylori (Schwabe and Paffrath 1996), (5) strong price reduction, (6) first choice in guideline for community-acquired pneumonia (Höffken et al. 2005). Key events for amoxicillin-clavulanic acid are (7) patent expiration and strong price reduction, (8) first choice in guideline for complicated community-acquired pneumonia (Ewig et al. 2016), (9) indication for recurrent sinusitis and otitis media (Ewig et al. 2021)

Aminopenicillins were not as important in the past as they are today. Over the time, they gained prominence in medical guidelines, replacing penicillins and becoming the most significant substance group. The rapid ascent began in 1995 with the introduction of amoxicillin in the treatment of gastric ulcers caused by H. pylori, which is depicted in Fig. 11 no. 4. After a price drop in 1997 (see Fig. 11 no. 5), prescriptions for amoxicillin and therefore aminopenicillins increased rapidly. Consequently, aminopenicillins became the most frequently prescribed substance group, with amoxicillin being the most prescribed substance since 2000. In 2003, the costs of aminopenicillins were lower than penicillins (see Fig. 11 no. 1). By 2005, amoxicillin was named the first choice in guidelines for the treatment of community-acquired pneumonia (Ewig et al. 2016) (see Fig. 11 no. 6). A distortion was caused by the inclusion of dental prescriptions since 2011 (see Fig. 11 no. 2). The COVID pandemic caused a dip in prescriptions (see Fig. 11 no. 3), which have returned to pre-pandemic levels since 2022. Amoxicillin-clavulanic acid started rising after its patent expiration and price drop around 2000 (see Fig. 11 no. 7). It has an extended spectrum compared to amoxicillin (Schwabe and Paffrath 2003). It is indicated for complicated community-acquired pneumonia since 2016 (Ewig et al. 2016) (see Fig. 11 no. 8) and for recurrent sinusitis and otitis media since 2020 (Ludwig et al. 2021) (see Fig. 11 no. 9). Since then, prescriptions have risen even more sharply, replacing amoxicillin in some cases.

### Cephalosporins

**Fig. 12:**
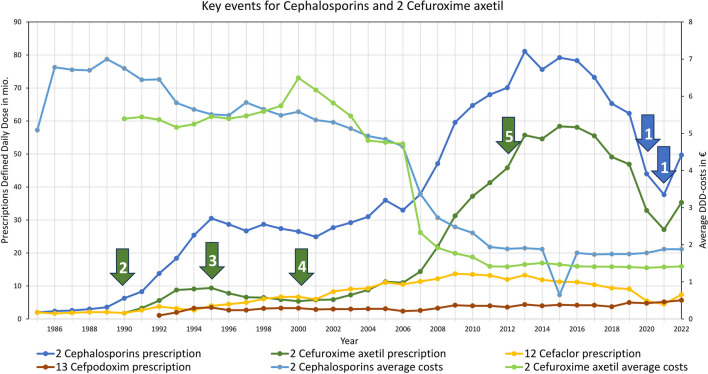
Key event for cephalosporins in general is (1) COVID-pandemic. Key events for cefuroxime axetil are (2) market launch and being more effective against gram-negative pathogens than cefaclor, (3) price drop of cefaclor, (4) patent expiration and strong price reductions, (5) no recommendation anymore for community-acquired pneumonia (Bätzing-Feigenbaum et al. 2016)

Cephalosporins were rarely used in the beginning. This can be explained by their high costs. In 1990, cefuroxime axetil was launched, which can be seen in Fig. 12 no. 2. As this substance was considerably more effective against gram-negative pathogens than cefaclor, its prescriptions rose. By 1995, cephalosporins were prescribed more frequently that fluoroquinolones. The sharp decline in the costs of cefaclor in 1995 led to a preference for it over cefuroxime axetil, resulting in a subsequent decrease in prescriptions (see Fig. 12 no. 3). In 2000, the expiration of the patent for cefuroxime axetil led to reduced costs and increased prescriptions (see Fig. 12 no. 4). By 2005, cephalosporins were prescribed more often than penicillins. Following a substantial price drop of cefuroxime axetil in 2007, prescriptions saw a sharp increase, despite the already-known issue of poor oral bioavailability of cephalosporins. Cephalosporins have held the position of the second most prescribed substance group since 2010. Between 2012 and 2015, cefuroxime axetil was no longer recommended in the guidelines for many indications (Bätzing-Feigenbaum et al. 2016) (see Fig. 12 no. 5). Nevertheless, prescriptions continued to rise, only starting to decline since 2017. The COVID pandemic caused a sharp decline in 2020 and 2021 (see Fig. 12 no. 1). In 2022, prescriptions returned to pre-pandemic levels (Ludwig et al. 2024), but with a further downward trend.

Many key events of cefaclor and cefpodoxime are related to advertising and significant price drops, as seen in Fig. S1 no. 1, 2 and 3. Cefpodoxime is still indicated for lues and gonorrhea as well as an alternative for urinary tract infections (see Fig. S1 no. 3 and 4). Compared to cefuroxime axetil, both drugs play a subordinate role.

### Macrolides and lincosamides (clindamycin)

**Fig. 13:**
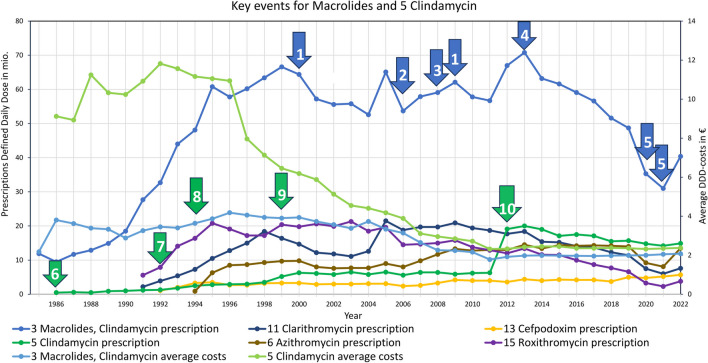
Key events for macrolides in general are (1) adverse effects like above-average gastrointestinal problems e.g. pseudomembranous colitis in 1998 (Schwabe and Paffrath 1999), exacerbation of myasthenia gravis (Cadisch et al. 1996) and stimulation of bacterial resistance (Baquero, 1999), as well as a high number of drug interactions and the risk of cardiac arrhythmias in 2009 (Simkó et al. 2008); (2) STIKO-recommendation for vaccination against pneumococci (Schwabe and Paffrath 2008), (3) increased resistance rates for pneumococci and A streptococci (GERMAP 2008, 2016), (4) falling resistance and number of infections due to vaccination (Schwabe and Paffrath 2014), (5) COVID-pandemic. Key events for clindamycin are (6) indication of osteomyelitis and being the first choice for legionellosis and pertussis (Schwabe and Paffrath 1987), (7) indication for anaerobic infections (Schwabe and Paffrath 1993), (8) patent expiration and strong price drop, (9) indication for severe staphylococcal infections (Schwabe and Paffrath 2000), (10) strong distortion due to the inclusion of dental prescriptions

Macrolides and clindamycin were already a component in the treatment of bacterial infections in 1985 and gained importance over the years. Clindamycin was indicated for osteomyelitis, legionellosis and pertussis (Schwabe and Paffrath 1987). This is shown in Fig. 13 no. 6. Additional indications were added in the following years (see Fig. 13 no. 7 and 9). Prescriptions of macrolides rose very sharply, primarily due to the launch of new drugs like roxithromycin and a price drop of clindamycin after the expiration of its patent in 1994 (see Fig. 13 no. 8). However, as more and more adverse effects became known (see Fig. 13 no. 1) and resistance increased (GERMAP 2008, 2016) (see Fig. 13 no. 3), prescriptions for macrolides decreased. In 2006, the STIKO recommended vaccination against pneumococci (Schwabe and Paffrath 2008) (see Fig. 13 no. 2), leading to a reduction in resistance and infections (Schwabe and Paffrath 2014) (see Fig. 13 no. 4). Consequently, fewer macrolides had to be prescribed. This continuous decrease is strongly distorted by the inclusion of dental prescriptions in clindamycin since 2012 (see Fig. 13 no. 10). A sharp drop due to the COVID pandemic can be seen in 2020 and 2021 (see Fig. 13 no. 5).

Azithromycin had an extended spectrum compared to other macrolides at its market launch, as seen in Fig. S2 no. 1. Initially, prescriptions increased, but quickly reached a plateau due to side effects and rising bacterial resistance (see Fig. S2 no. 2, 3, 5). With the introduction of generics, accompanied by falling costs, prescriptions rose to a slightly higher plateau (see Fig. S2 no. 5). Clarithromycin is a component in the therapy of gastric ulcers caused by H. pylori (see Fig. S2 no. 6), but bacterial resistance developed (Bluemel et al. 2020) (see Fig. S2 no. 8 and 9). After a price reduction took place (see Fig. S2 no. 7), prescriptions increased before falling again. Roxithromycin had therapeutic advantages due to a low daily dose (see Fig. S2 no. 10), which led to rising prescriptions. A price drop had little impact (see Fig. S2 no. 11). Over the years, roxithromycin lost its impact.

### Tetracyclines

Tetracyclines were the most prescribed group in 1985, but have declined since 1994. Despite their very low average DDD-costs (see Fig. S3 no. 2 and 6), tetracyclines hold little significance today. This change was caused by a significant development of bacterial resistance (see Fig. S3 no. 1 and 8) (Schwabe and Paffrath 1992) and a rising popularity of amoxicillin (see Fig. S3 no 3). Doxycycline is the most frequently used tetracycline because of therapeutic benefits (see Fig. S3 no. 5). Currently, these drugs are used for chlamydial urethritis, skin and soft tissue infections as well as in the ENT area. They are effective against MRSA and Q fever (see Fig. S3 no. 7, 9 and 11) (Ludwig et al. 2024). There is potential in off-label use for psychiatric diseases, although this could have a negative impact on the development of resistance (see Fig. S3 no. 10) (Chaves et al. 2021). In 2020 and 2021, the COVID pandemic caused a slight dip in prescription numbers (see Fig. S3 no. 4).

### Other drugs

The group of other antibacterial drugs is a very heterogenous substance group (Fig. S4). Over the years, this group has gained slightly more impact, mainly in the field of urinary tract infections. Nitrofurantoin is indicated for uncomplicated urinary tract infections (Kniehl et al. 2010) (see Fig. S4 no. 4). Due to adverse effects, it should be used with caution (see Fig. S4 no. 3 and 5). Since the market launch of pivmecillinam (see Fig. S4 no. 6), the number of prescriptions is falling slightly.

### Penicillins

Penicillins were often used until 1995. Afterwards, they were replaced by aminopenicillins (Fig. S5 no. 1, 2 and 5). A distortion in the data was caused by the inclusion of dental prescriptions (see Fig. S5 no. 3), a COVID dip is recognizable too (see Fig. S5 no. 4). Pivmecillinam is an exception to the downward trend in penicillins. Since its market launch (see Fig. S5 no. 6) it has shown an increasing trend. This is due to its indication for uncomplicated cystitis (see Fig. S5 no. 7) and effectiveness against gram-negative bacterial (Fuchs and Hamprecht 2019) (see Fig. S5 no. 7). Unfortunately, there are serious indications of a rapid development of resistance in bacterials causing urinary tract infection (Stoltidis-Claus et al. 2023) (see Fig. S5 no 8).

### Sulfonamides

Sulfonamide-trimethoprim (Fig. S6), lost its significance due to strongly rising bacterial resistance (Karlowsky et al. 2002; Ludwig et al. 2024). In the beginning, it was used for many indications (see Fig. S6 no. 1 and 2). Because of rising bacterial resistance (see Fig. S6 no. 3, 5) and the popularity of amoxicillin (see Fig. S6 no. 4), indications have been withdrawn (see Fig. S6 no. 6). In recent years, an important indication has been an alternative for MRSA (Cadena et al. 2011) (see Fig. S6 no. 7). The COVID pandemic had very little impact on its prescriptions (see Fig. S6 no. 8).

### Fluoroquinolones

Fluoroquinolones, especially ciprofloxacin, had once a very wide spectrum and many indications (Fig. S7 no. 6). As fluoroquinolones have always been a comparatively expensive drug class, the popularity of amoxicillin led to a particularly sharp decline (see Fig. S7 no. 2). After the patent expiry of ciprofloxacin, a renewed increase was recorded (see Fig. S7 no. 7). Due to many adverse effects, there were withdrawals of many drugs (see Fig. S7 no. 1). A strong increase in resistance (see Fig. S7 no. 3) caused the revocation of many indications (see Fig. S7 no. 4). The COVID pandemic had little impact on prescriptions (see Fig. S7 no. 5).

### Costs as major driver of prescriptions

**Fig. 14:**
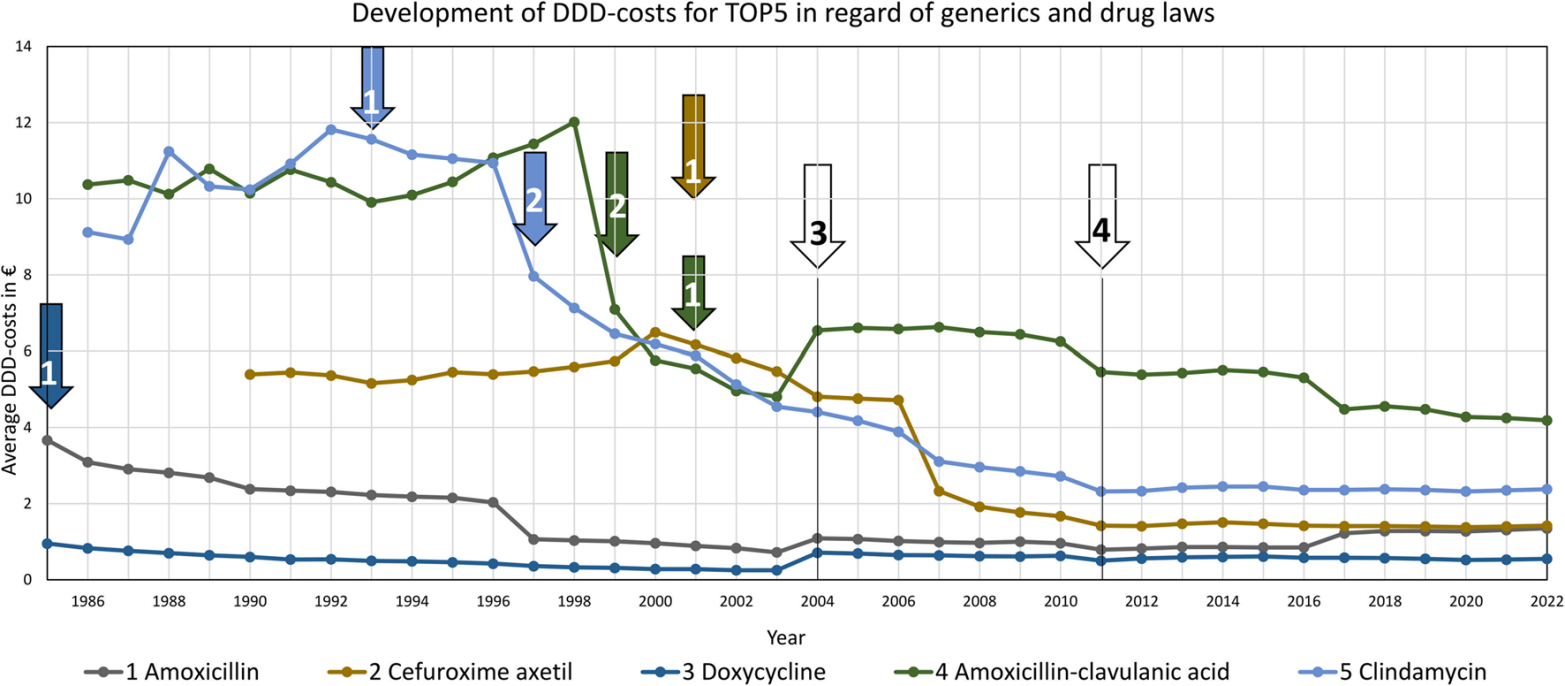
Key events are (1) introduction of generics, (2) significantly lower-priced preparation available, (3) GMG (Schwabe and Paffrath 2007), (4) AMNOG (AOK 2023)

Costs have a very strong influence on prescriptions. Falling costs go hand in hand with rising prescriptions. Prescriptions often only increase after a significant price reduction. For all TOP15 drugs, sinking costs cause rising prescriptions, which is depicted in Table 1. In contrary, a significant price increase leads to decreasing prescriptions. A typical cost curve starts at a comparatively high level with a slightly rising plateau. After a certain period, there is a sudden, rapid drop with an approach to a significantly lower level. This is often induced by the introduction of generics or significantly cheaper preparations. Figure 14 shows such a trend for the TOP5, but it can also be recognized for many TOP15 (see Table 1).

**Table 1:** Relation between sinking costs and rising prescriptions. Depicted are the absolute and relative change in prescriptions (DDD) and costs of the TOP15 (reference year: 2022) in the years around the largest relative cost change. The largest changes in costs were differentiated into rising (increase) vs. falling (reduction) DDD-costs

ranking of TOP15	antibacterial drug	year of the largest relative cost change	prescriptions in the year before cost change	prescriptions in the year after cost change	relative change in DDD costs	absolute change in DDD costs	relative change in prescriptions	absolute change in prescriptions
**1**	Amoxicillin	1997 (reduction)	29.2 mio (1996)	54.3 mio (1997)	− 47.8%	− 1.0 €	+ 86.0%	+ 25.1 mio
**2**	Cefuroxime axetil	2007 (reduction)	11.0 mio (2006)	14.4 mio (2007)	− 50.6%	− 2.4€	+ 23.6%	+ 3.4 mio
**3**	Doxycycline	2004 (increase)	56.3 mio (2003)	51.3 mio (2004)	+ 284%	+ 0.5 €	− 9.8%	− 5.0 mio
**4**	Amoxicillin clavulanic acid	1999 (reduction)	1.5 mio (1998)	2.6 mio (1999)	− 59.1%	− 5.1 €	+ 73.3%	+ 1.1 mio
**5**	Clindamycin	1997 (reduction)	2.9 mio (1996)	3.0 mio (1997)	− 27.1%	− 3.0 €	+ 3.5%	+ 0.1 mio
**6**	Azithromycin	2007 (reduction)	8.0 mio (2006)	9.8 mio (2007)	− 21.9%	− 1.1 €	+ 22.5%	+ 1.8 mio
**7**	Phenoxymethyl-penicillin	2004 (increase)	31.9 mio (2003)	27.7 mio (2004)	+ 83.2%	+ 0.8 €	− 13.2%	− 4.2 mio
**7**	Sulfonamide-Trimethoprim	2004 (increase)	22.5 mio (2003)	22.2 mio (2004)	+ 367.4%	+ 1.2 €	-1.3%	− 0.3 mio
**9**	Nitrofurantoin	2011 (reduction)	9.2 mio (2010)	10.4 mio (2011)	− 19.8%	− 0.2 €	+ 13.0%	+ 1.2 mio
**10**	Ciprofloxacin	2003 (reduction)	6.9 mio (2002)	8.8 mio (2003)	− 31.3%	− 3.1 €	+ 27.5%	+ 1.9 mio
**11**	Clarithromycin	2005 (reduction)	12.6 mio (2004)	21.5 mio (2005)	− 30.9%	− 1.3 €	+ 70.6%	+ 8.9 mio
**12**	Cefaclor	1995 (reduction)	2.7 mio (1994)	4.0 mio (1995)	− 22.2%	− 1.7 €	+ 48.2%	+ 1.3 mio
**13**	Cefpodoxime	2007 (reduction)	2.4 mio (2006)	2.6 mio (2007)	− 30.4%	− 1.6 €	+ 8.3%	+ 0.2 mio
**14**	Pivmecillinam	2019 (reduction)	0.6 mio (2018)	1.4 mio (2019)	− 2.6%	− 0.1 €	+ 153.6%	+ 0.8 mio
**15**	Roxithromycin	2001 (reduction)	19.8 mio (2000)	20.6 mio (2001)	− 17.3%	− 0.6 €	+ 4.0%	+ 0.8 mio

For 2004, an increase is depictable for several drugs (see Fig. 14). It is very likely that these distortions are caused by drug laws. The *GKV-Modernisierungsgesetz* (*GMG*, see Fig. 14 no. 3*)* was enacted around 2004, introducing a uniform pharmacy dispensing fee (Schwabe and Paffrath 2007). Since antibacterial drugs have lower daily costs compared to other drug classes, the DDD costs increased. Conversely, the Arzneimarktneuordnungsgesetz (AMNOG, see Fig. 14 no. 4) of 2011 supported discount contracts and price reductions in favour of the health insurance companies (AOK 2023). Overall, several laws were enacted to alleviate the financial burden of escalating drug costs, but the effect on DDD-costs is not clear in many cases.

In general, all drugs show a substantial drop in price over time (Table 1). Exceptions are newly introduced drugs such as pivmecillinam, for which patent protection has not yet expired. For these drugs, a price decline is to be expected in the future too. Many drugs such as amoxicillin, cefuroxime axetil or amoxicillin clavulanic acid show a sharp sudden drop in price with a strong percentage increase of prescriptions in the year of the reduction. In the case of other drugs, the decrease in costs shows a steady reduction, resulting in a steady increase in the number of prescriptions. The larger the relative costs fall, the greater is the impact on the number of prescriptions. For some drugs, the GMG in 2004 resulted in such a sharp price increase. This event did not lead to an immediate decrease in prescriptions for the particularly affected drugs, as a slight increase in costs was recorded for many preparations. In the medium term, however, there were significant decreases in prescriptions, caused by a permanently higher price level. Increases in DDD-costs usually do not result in an immediate sharp drop, but lead to a long-term decline in prescriptions. As a result, the immediate impact of relative cost increases on the relative change in prescriptions is low.

Antibacterial drugs influence each other. Trends such as replacement, e.g. aminopenicillins vs. penicillins (see Figs. 3 and 5) and constant competition, e.g. within the macrolides (see Fig. S3), can be recognized. This means that events affecting one drug can also have an indirect impact on others.

### Bacterial resistance, adverse effects and COVID-19

Further influencing factors are apparent. The development of bacterial resistance has led to a decline due to diminishing therapeutic effectiveness. This applies to fluoroquinolones and sulfonamidee-trimethoprim. Information about adverse effects and indications can help ensure that a drug is prescribed according to the indication. This was particularly noticeable around the year 2010 concerning the adverse effects of fluoroquinolones (see Fig. S7) and the poor oral efficacy of cefuroxime axetil (see Fig. 10). The number of infection cases also plays a role. This was evident during the COVID-19 pandemic, which led to a drop in prescriptions for almost all drug classes (see Fig. 3).

#### Importance of general practitioners

Only a few groups prescribe the main share of antibacterial drugs (see Figs. 9 and 10 and S8). In 2022, the primary contributors were general practitioners, internists working as general practitioners and dentists. These specialist groups are part of the primary care and serve as the initial point of contact for acute illnesses. Their prescription rates are significantly influenced by the overall incidence of infection, as exemplified by the huge impact of the COVID pandemic, which precipitated a temporary decline in prescriptions. In recent years, general practitioners have been prescribing fewer antibacterial drugs, which is a positive trend. In general, professionals in primary care are important points of intervention.

There are a multitude of specialized fields such as ophthalmologists, orthopedics, cardiologists, nerve specialists, nephrologists, among others. Collectively, these specialists contribute only a small fraction of prescriptions. This is due to the fact that these doctors only treat diseases specifically and purposefully for their area of expertise. Consequently, they are less influenced by the general incidence of infection. The impact of the COVID pandemic on their prescription rates has been minimal. In recent years, many groups have experienced slight increases in prescription rates at relatively low levels. This trend may be attributed to advancements in diagnostic techniques and treatment options for diseases, as well as demographic changes leading to a higher proportion of elderly and morbid patients.

### Comparison of antibacterial drug consumption in the European Union

**Fig. 15:**
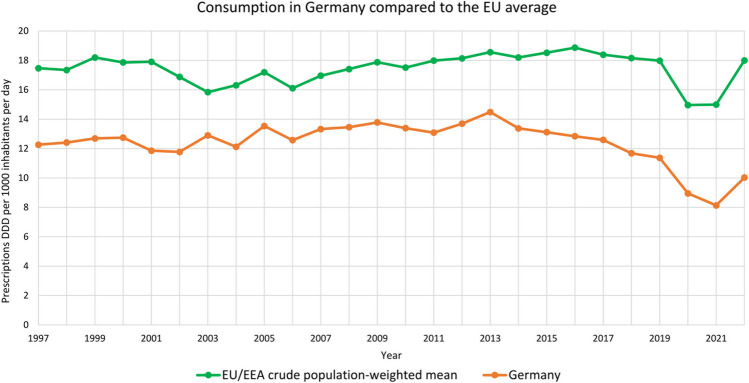
Consumption of antibacterial drugs in Germany compared to the EU average, ATC group J01 in the community (primary care) sector, prescriptions Defined Daily Dose per 1000 inhabitants per day from 1997–2022 (ECDC 2024)

The comparison of antibacterial drug consumption in the European Union is essential for evaluating prescription behaviour. When examining antibacterial drug prescriptions in other EU countries, Germany ranks in the bottom third (ECDC 2020). For many years, there has been a clear north-south and west-east divide in terms of prescriptions: while northern Europe exhibits low consumption, southern Europe demonstrates very high consumption. This shift is not only evident in consumption but is also apparent in the development of bacterial resistance (ECDC 2023b). Nevertheless, the consumption of Germany and the European Union follow the same trend. This is visualized in Fig. 15.

In countries with comparable low consumptions, a high proportion of penicillins and older generations of cephalosporins are prescribed. In contrast, countries with high consumptions tend to use many beta-lactams with an extended spectrum and a comparatively high proportion of newer generations of macrolides, fluoroquinolones, and cephalosporins (ECDC 2020; de With et al. 2004).

Consumption in the outpatient sector has been declining in many countries in recent years (ECDC 2023a). Due to the COVID-19 pandemic, a significant decline was observed in almost all countries except Bulgaria. Serious developments include the sharp increase in the use of reserve drugs, the need to use alternatives due to supply bottlenecks, and a declining rate of new antibacterial drugs developed to market maturity.

#### Limitations

The analysis in this study is based on data from the Arzneiverordnungsreport. Since only outpatient prescriptions of the GKV system are included, no assessment can be made regarding prescriptions in hospitals or via private health insurance. The evaluation depended on the design of the AVR chapter under consideration. Changes in the structure of the AVR sections over the years led to distortion in individual cases. As interactions and influences on developments are very complex, it cannot be guaranteed that all decisive events were always recognized. By focusing on the TOP15, it is possible that key events for some less often prescribed drugs have been overlooked. As the data is based on the development in Germany, it is not directly transferable to other countries. No differentiation was made about age or region.

## Conclusions

Regarding antibacterial drugs, there have been positive and problematic developments in recent years. The emergence of bacterial resistance has restricted the use of many drug groups, and there is a growing shortage of supplies (BfArM 2023). Positive trends include the decrease in overall prescriptions, with drugs being prescribed more in line with indications. When costs are very low, drugs might be prescribed excessively and not be appropriate for their indication, as applies to cefuroxime axetil.

Antibacterial drugs are mainly prescribed in primary care settings. Encouragingly, there has been a downward trend in antibacterial drug prescriptions by general practitioners in recent years. Recognizing the pivotal role of primary care professionals, interventions targeted at this level hold substantial promise for improving overall antibacterial stewardship and combating antimicrobial resistance. Implementing improvement measures within these groups will have the greatest effect in the overall development.

The strong influence of costs on the number of prescriptions is very remarkable (see Table 1). Decreasing costs, often caused by the introduction of generics, lead to a significant increase in prescriptions. This prompts the question whether costs are the strongest influencing factor, despite the expectation that efficacy or the development of bacterial resistance would be more relevant.

Moreover, falling costs may pose an increasing risk of supply bottlenecks. While decreased prices incentivizes professionals to prescribe a certain drug more frequently, the profit margin for pharmaceutical companies diminishes. Consequently, there is little incentive to increase production capacity, resulting in a discrepancy between supply and demand.

In comparison to other European countries, Germany ranks in the lower third regarding prescriptions (ECDC 2020). Overall, most countries in the EU have seen a decline in prescriptions in recent years (ECDC 2023a). Consumption in Germany, compared to the EU average, follows a similar trend (see Fig. 15). Given the discernible north-south and west-east shift is recognizable with higher consumption and bacterial resistance in southern and eastern regions (ECDC 2023b), it is likely that developments in other countries will also impact Germany.

Studies have shown that there is still potential for reducing antibacterial drug consumption in Germany (Kern and Kostev 2021). Respiratory tract infections are often caused by viruses, for which antibacterial drugs have no effect. To clarify this fact, it makes sense to use the term “antibacterial drugs” instead of “antibiotics” (Seifert and Schirmer 2021). In general, more attention should be paid to good doctor-patient communication and clear guidelines so that important changes considered in everyday clinical practice.

### Take-home messages

Our study clearly shows that price (DDD costs) is a very powerful tool to regulate antibacterial drug prescriptions. Substantial increases in current prices for antibacterial drugs may achieve four goals:

1. More careful and critical prescription of antibacterial drugs for unproven bacterial infections that in many cases are actually viral infections.
2. Reduced risk of bacterial resistance development with reduced pressure to develop new antibacterial drugs.
3. Increased incentive for drug companies to produce antibacterial drugs in Europe instead in India and China with reduced risk of supply shortages in Europe.
4. Reduced risk of adverse effects caused by antibacterial drugs.

## Electronic supplementary material

Below is the link to the electronic supplementary material.

Supplementary Material 1 (DOCX 1.17 MB)

## Data Availability

All source data for this study are available upon reasonable request from the authors.

## Abbreviations

AVR: Arzneiverordnungsreport (Drug Prescription Report regarding the GKV system of Germany)
AMNOG: Arzneimarktneuordnungsgesetz (Pharmaceutical Market Reorganization Act), 2011
DDD: Defined Daily Dose
GKV: gesetzliche Krankenversicherung (statutory health insurance)
GMG GKV: Modernisierungsgesetz (Statutory Health Insurance Modernization Act), 2004
MRSA: methicillin-resistant Staphylococcus aureus
TOP15: group of the 15 most prescribed antibacterial drugs (based on the AVR 2023)
TOP5: group of the 5 most prescribed antibacterial drugs (based on the AVR 2023)
WidO: wissenschaftliches Institut der AOK (scientific institute of the AOK)
