# Supplementary material for: Costs are a major driver of antibacterial drug prescriptions in Germany: market analysis from 1985 to 2022
Source: Naunyn Schmiedebergs Arch Pharmacol. 2024 Jun 6;397(11):8785–801. doi: 10.1007/s00210-024-03171-y (PMC11522090; doi:10.1007/s00210-024-03171-y)

**Costs are a major driver of antibacterial drug prescriptions in Germany: market analysis from 1985-2022**

**Lilly Josephine Bindel and Roland Seifert**

Supplemental Figures

***Fig. S1****: Key events for cefaclor are (1) intensified advertising for fixed-price exempt drugs (Schwabe and Paffrath 1992), (2) significant price drop. Key events for cefpodoxim are (3) patent expiration and price drop, (4) indication as an alternative for urinary tract infections (Kniehl et al. 2010), (5) use in therapy of lues and gonorrhea (Schwabe and Ludwig 2020).*


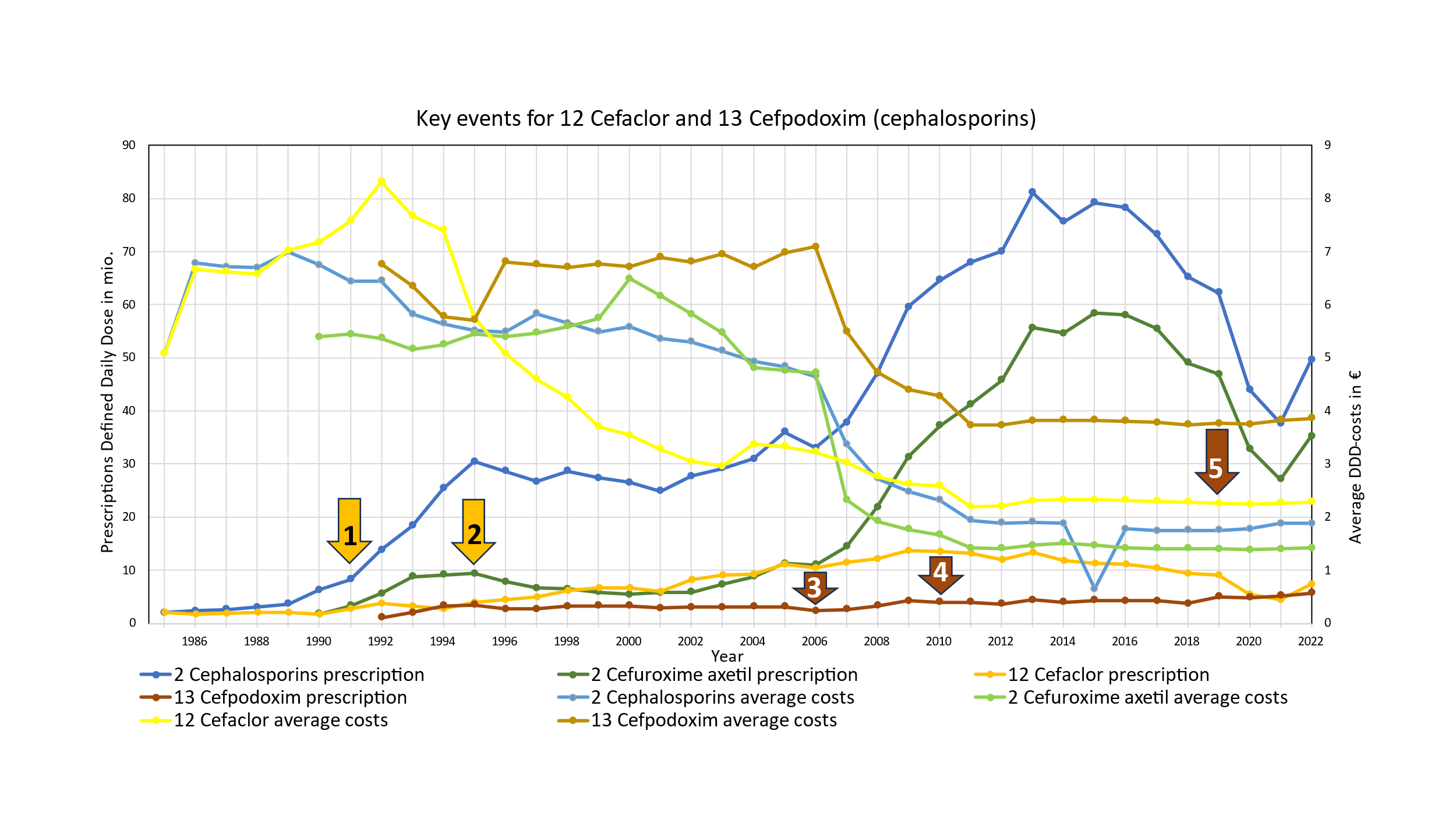


***Fig. S2****: Key events for azithromycin are (1) market launch, extended spectrum than other macrolides, pharmacokinetic advantages (Schwabe and Paffrath 1995), (2) long duration of therapy problematic due to animal testing (Schwabe 1997), (3) contraindication pregnancy (Schwabe and Paffrath 2004), (4) promoted development of bacterial resistance because of long subinhibitoric concentration (Baquero 1999; Schwabe and Paffrath 2003), (5) patent expiration and price drop.
Key events for clarithromycin are (6) component in triple-therapy of gastric ulcers caused by H. pylori (Schwabe and Paffrath 1996), (7) patent expiration and price drop, (8) not being effective anymore against haemophilus (Schwabe and Paffrath 2006), (9) rising resistance of H. pylori (Bluemel et al. 2020).
Key events for roxithromycin are (10) low daily dose (Schwabe and Paffrath 1993), (11) patent expiration and price drop.*

*
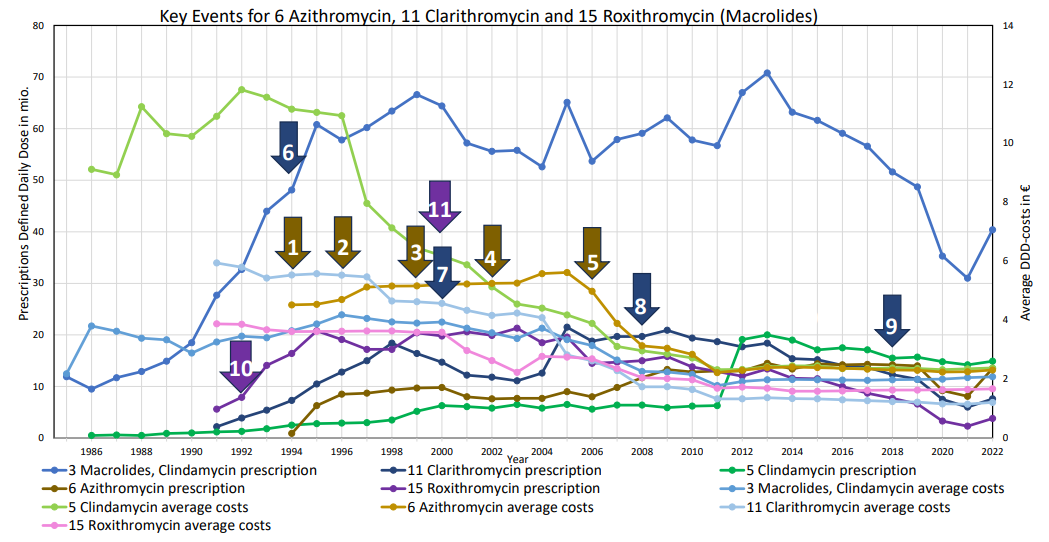
*

***Fig. S3****: Key events for tetracyclines in general are (1) rising resistance due to frequent use (Schwabe and Paffrath 1992), (2) cheapest substance group, (3) sharp increase in prescriptions of amoxicillin, (4) COVID-pandemic.
Key events for doxycycline are (5) therapeutic benefits to other tetracyclines (Schwabe and Paffrath 1985), (6) cheapest substance of TOP15, (7) first choice for chlamydial urethritis (Schwabe and Paffrath 2003), (8) rising resistance of E. coli (Schwabe and Paffrath 2004), (9) indicated for MRSA as well as skin and soft tissue infections (Schwabe and Paffrath 2010), (10) chance of new neuropsychiatric indications (Chaves et al 2021), (11) frequently used in ENT area, effective against Q fever (Ludwig, Mühlbauer, Seifert 2024).*

*
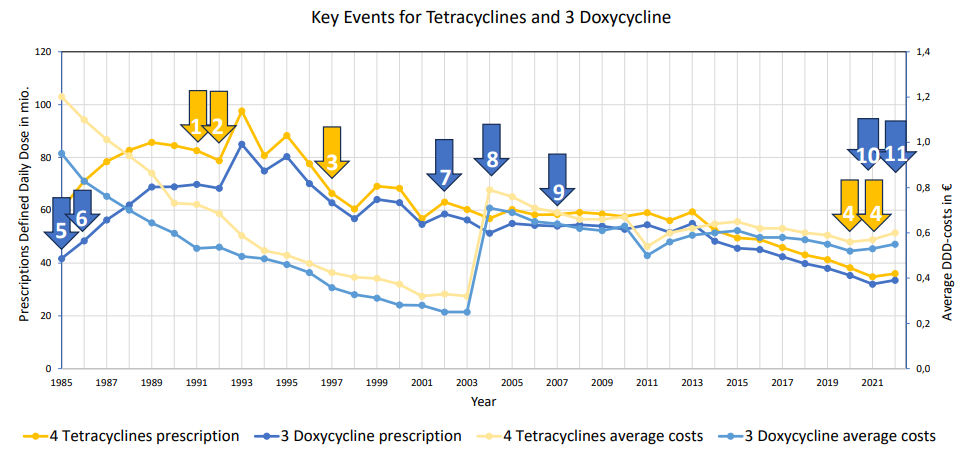
*

***Fig. S4****: Key events for other antiinfective chemotherapeutics are (1) sharp rise in prescription of fusafungin, (2) sharp decline in prescription of fusafungin.
Key events for Nitrofurantoin are (3) side effects (Schwabe and Paffrath 2008, 2010), (4) indication for uncomplicated urinary tract infections due to guideline (Kniehl et al. 2010), (5) cautious use recommended due to malformations in animal testings (Schwabe and Paffrath 2015), (6) market launch of pivmecillinam.*

*
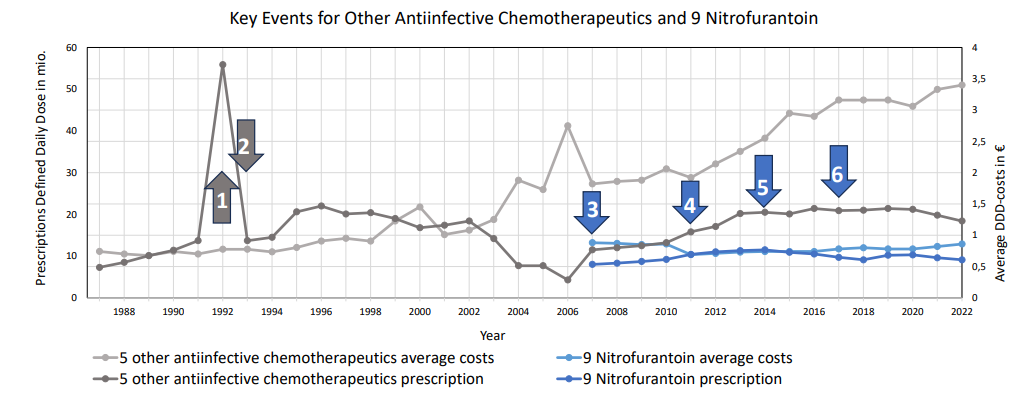
*

***Fig. S5****: Key events for penicillins in general are (1) sharply rising prescriptions of amoxicillin, (2) higher costs than aminopenicillins / amoxicillin, (3) distortion due the inclusion of dental prescriptions, (4) COVID-pandemic.
Key event for phenoxymethylpenicillin is (5) not anymore first choice in guidelines (replaced through amoxicillin) (Schwabe and Paffrath 2004).
Key events for pivmecillinam are (6) market launch, (7) indicated for uncomplicated cystitis (Schwabe et al. 2019), effective against ESBL-producing gram-negative bacterials (Fuchs et al. 2019), (8) alarming rapid development of bacterial resistance for urinary tract infections (Stoltidis-Claus et al. 2023).*


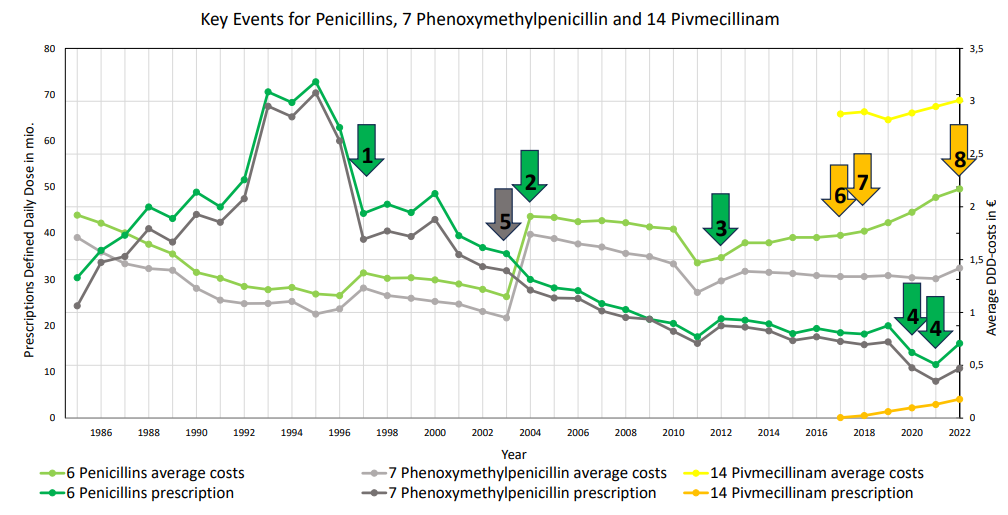


***Fig. S6****: Key events for sulfonamide-trimethoprim are (1) first choice for urinary tract infections (incl. short-time therapy) and salmonellosis, alternative for chronic bronchitis (Schwabe and Paffrath 1986); (2) first choice for pneumocystis-carinii-pneumonia (Schwabe and Paffrath 1993), (3) ineffective for strict anaerobiers and many pneumococcus (Schwabe 1997), (4) strong increase of prescriptions of amoxicillin, (5) rising resistance E. coli (Karlowsky et al 2002; Schwabe and Paffrath 2003, 2004, 2010; Paul-Ehrlich-Gesellschaft 2008), (6) no recommendation for treatment of empiric therapy for urinary tract infections due to high resistance rates of E. coli (Paul-Ehrlich-Gesellschaft 2008; Schwabe and Paffrath 2011), (7) alternative for treatment MRSA (Cadena et al. 2011), (8) COVID-pandemic.*


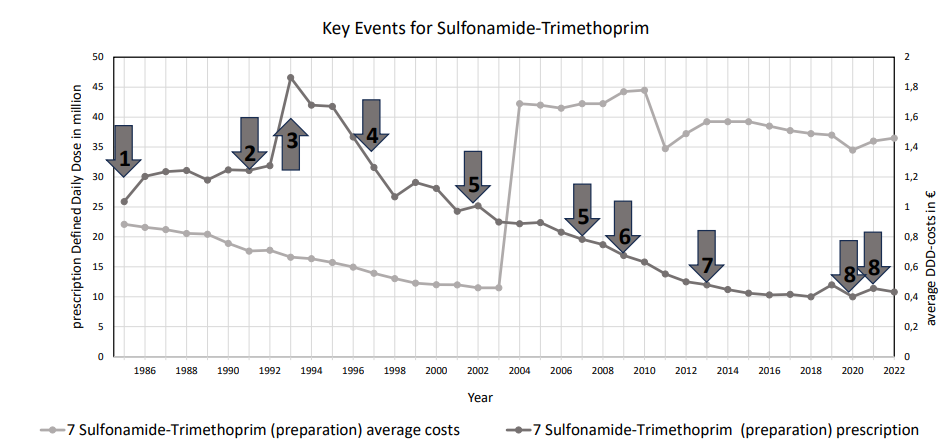


***Fig. S7****: Key events for fluoroquinolones in general are (1) withdrawal of various preparation from market due to side effects, (2) most expensive substance group and strong rise in prescriptions of amoxicillin, (3) rising bacterial resistance (Schwabe and Paffrath 2003; Paul-Ehrlich-Gesellschaft 2016), (4) no indication anymore for community-acquired pneumonia (Schwabe and Paffrath 2008), (5) COVID-pandemic.
Key events for ciprofloxacin are (6) extended spectrum and pharmacokinetic advantages to older fluoroquinolones; indication for urinary tract infections, pneumonia, skin and abdominal infections (Schwabe and Paffrath 1988), (7) patent expiration and price drop.*

*
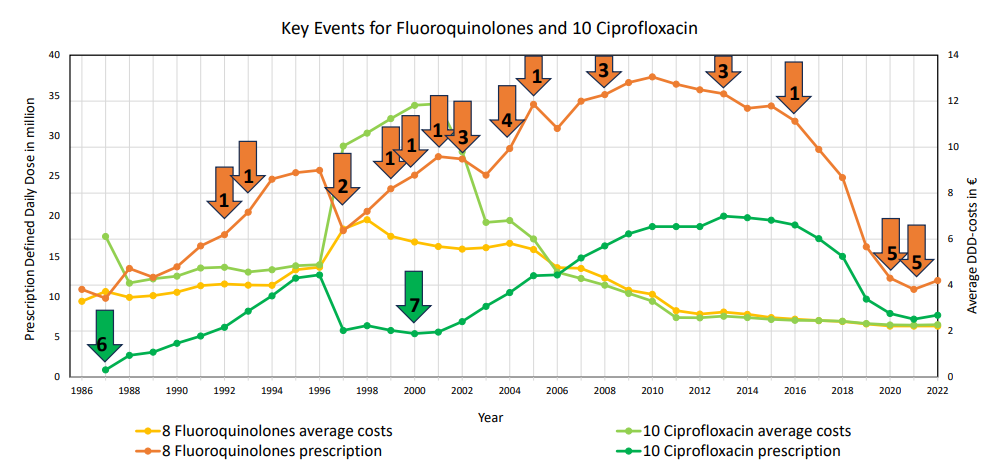
*

***Fig. S8****: Relative prescriptions of antibacterial drugs from 2008-2022, broken down by specialist groups, dental prescriptions are excluded (from 2012-2022)*


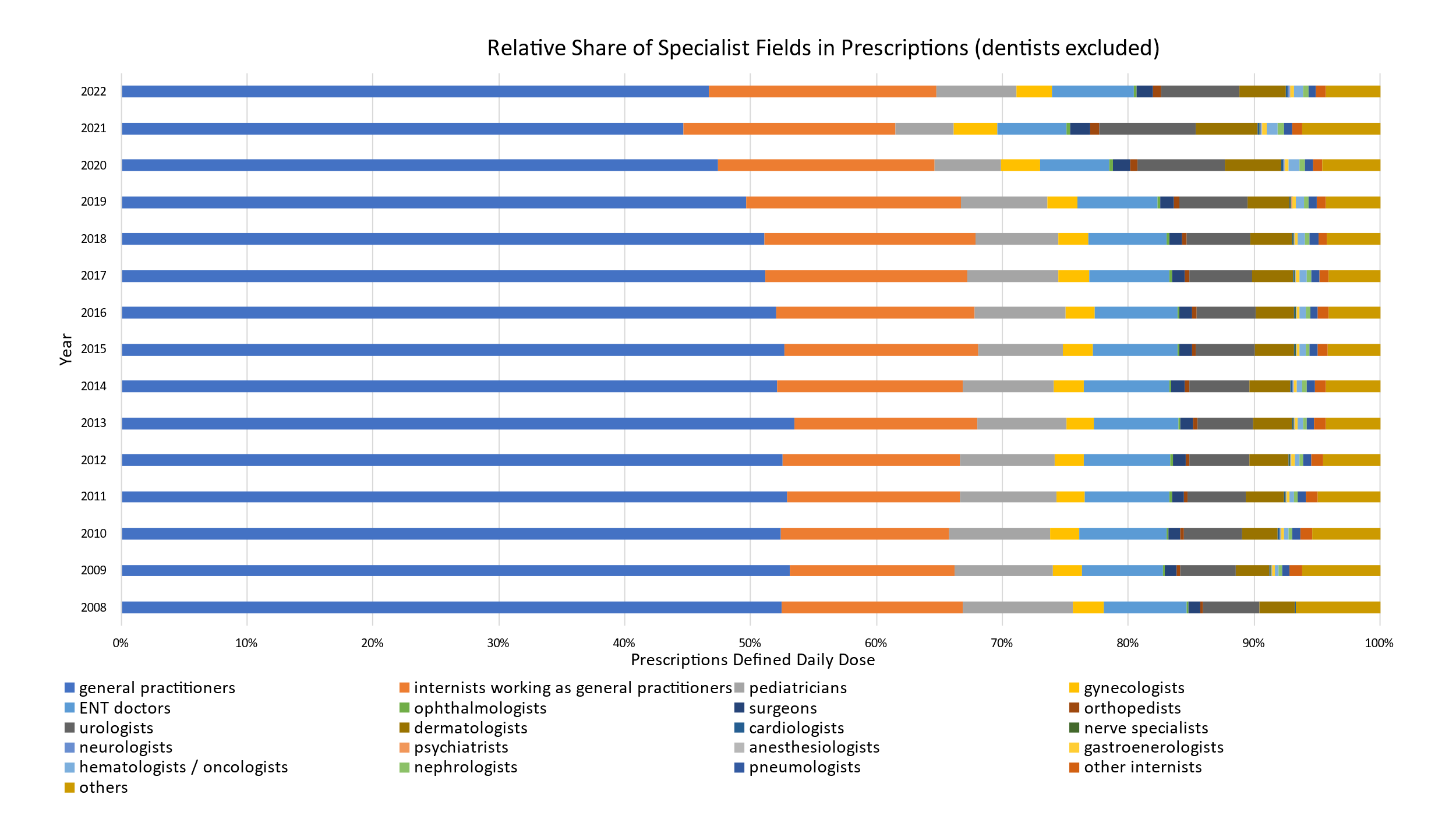

Supplement: Supplementary file 1 — Supplementary Material 1 (DOCX 1.17 MB) [file 210_2024_3171_MOESM1_ESM.docx]
